# Validation and clinical correlates of the Turkish version of the methotrexate intolerance and severity assessment (MISA) questionnaire in rheumatoid arthritis

**DOI:** 10.1007/s11739-025-03983-7

**Published:** 2025-07-18

**Authors:** Haluk Cinakli, Gülay Alp, İdil Kurut Aysin, Servet Akar, Dilek Solmaz

**Affiliations:** Division of Rheumatology, Department of Internal Medicine, Faculty of Medicine, İzmir Katip Çelebi University, İzmir, Turkey

**Keywords:** Rheumatoid arthritis, Methotrexate intolerance, MISA questionnaire

## Abstract

The ‘‘Methotrexate Intolerance and Severity Assessment in Adults’’ (MISA) questionnaire was developed in patients with rheumatoid arthritis (RA) to better define methotrexate (MTX)- related intolerance. This study aims to translate and validate the MISA questionnaire in Turkish. A total of 130 RA patients who used MTX for at least three months were included in the study. The Turkish version of the MISA was developed following the recommended guidelines for cross-cultural adaptation. The validity of the test was assessed using Cronbach’s α agreement. Test–retest reliability was tested with a 2-way random effect intra-class correlation coefficient (ICC). Intolerance by physician's decision was accepted as the gold standard. The Kappa test analyzed the agreement between intolerance according to the physician and intolerance according to the MISA questionnaire. Of all patients, 105 (80.8%) were female, with a mean age of 55.8 ± 10.50 years. The Cronbach’s α value of the MISA questionnaire was 0.831 and had a good internal consistency. The total score of the MISA questionnaire was 1.7 ± 3.39. Test–retest assessments were conducted with a group of 32 patients. The test–retest ICC value was excellent, with a value of 0.894. There were 38 (29.2%) patients with MTX intolerance, according to the physician, and 55 (42.3%) patients with intolerance, according to the MISA questionnaire. The kappa agreement between physician-identified intolerance and intolerance identified by the MISA questionnaire was 0.721, indicating significant agreement. The Turkish version of the MISA questionnaire has been found valid, reliable, reproducible, and easily applicable.

**Supplementary Information:**

The online version contains supplementary material available at 10.1007/s11739-025-03983-7.

## Introduction

Rheumatoid arthritis (RA) is a chronic inflammatory condition primarily affecting synovial joints. This process can lead to damage in both the joints and other organs, particularly the lungs. Consequently, early recognition of the disease and prompt treatment are crucial [1]. Methotrexate (MTX) has been a staple in treating RA for approximately 50 years and remains a central role in therapy today [2, 3]. Despite its effectiveness and low cost, the side effects that occur during its use can lead to limitations in the use of MTX [4, 5]. It can cause common gastrointestinal (GI) side effects such as nausea, vomiting, and dyspepsia, as well as undesirable effects like headaches and dizziness. This situation is considered **“MTX intolerance”** and is reported as the most common reason for discontinuing the drug [6, 7].

In a systematic review, MTX persistence was found to be 50% to 94% in the first year, while it was reported as 25% to 79% over five years [8]. Identifying MTX intolerance and its severity can significantly contribute to its appropriate management while monitoring the patient. This process enhances patient compliance and disease control by enabling early and timely medication changes. Simultaneously, it can provide insights into which patients may develop intolerance and facilitate treatment selection [9]. This evaluation is possible using evaluation tools that are valid and reliable in the disease population.

The ‘‘Methotrexate Intolerance Severity Score (MISS)’’ questionnaire was developed by Maja Bulatovic et al. in 2011 in patients with juvenile idiopathic arthritis (JIA) to detect MTX intolerance [10]. There was still a need for adult patients. Based on the MISS questionnaire, Deeksha Vijaykumar et al. developed and validated the ‘‘Methotrexate Intolerance and Severity Assessment (MISA)’’ questionnaire to detect MTX intolerance in adult patients with RA in 2021. The MISA questionnaire consists of a total of 10 questions. According to the MISA questionnaire, patients with a score of 1 and above were considered intolerant to MTX. At the same time, the MISA-Cross Product (CP) score was developed to measure the severity of intolerance. Those with a MISA-CP score of 4 and above were also considered to have moderate to severe MTX intolerance. The internal consistency of the original version of the MISA questionnaire was found to have a Cronbach’s alpha of 0.641. In the receiver operating characteristic analysis, the area under the curve value was relatively high at 0.904 [95% Confidence interval (CI)] [11].

The primary purpose of this study is to examine the validity, reliability, and psychometric properties of the Turkish version of the MISA questionnaire. The secondary aim is to determine the relationships between the results obtained from the Turkish version of the MISA questionnaire and the clinical characteristics of RA patients.

## Material and methods

### Study population

This study included 130 RA patients who applied to the university rheumatology clinic between January 2022 and March 2022. Ethics committee approval was obtained from the local ethics committee prior to the study (Decision number: 2021-GOKAE-0662). The study was conducted in accordance with the Declaration of Helsinki, and written informed consent was obtained from all patients.

The patients had to meet the following criteria: a diagnosis of RA according to the 2010 American College of Rheumatology/European League Against Rheumatism classification criteria, volunteering to participate in the research and using oral or subcutaneous MTX treatment for at least three months. The exclusion criteria included cognitive impairments and a history of gastrointestinal cancer.

Information, such as patient code, gender, age, education period, occupation, height, weight, body mass index (BMI), marital status, disease duration, disease activity and quality of life scales, comorbidities, laboratory values, radiographic features, and medications used by all patients was recorded. The presence of MTX intolerance, as determined by the clinician, was considered the gold standard.

### Development of the Turkish version of the MISA questionnaire

It was planned to include at least 100 patients over the age of 18 who were diagnosed with RA using MTX. The number of patients was calculated at least 10 per questionnaire item [12]. Before starting the study, permission was obtained from the author of the original MISA questionnaire. The guidelines recommended by Beaton et al. were followed in translating and validating the MISA questionnaire into Turkish [13]. First, the original questionnaire was translated into Turkish by two native speakers: one psychologist and one English teacher, both of whom have advanced proficiency in English. Afterward, these two translations were merged into a single standard translation and then translated back into English by two native Turkish-speaking English teachers. Following that, a preliminary version of the questionnaire was developed by a committee. A pilot study was conducted using this version with 30 healthy individuals. Based on the pilot study, necessary adjustments were made without altering the meaning of the questionnaire, achieving cultural adaptation (Supplementary Table 1). The final version, created after the pilot version, was administered to 130 patients with RA using MTX. To assess test–retest reliability, the questionnaire was repeated 3–14 days after the initial test with 32 individuals who had previously completed the questionnaire.

### Statistical analysis

Statistical analysis of all the data obtained was performed using SPSS 22.0 (IBM Statistical Package for the Social Sciences software version 22.0). The normal distribution of numerical data was evaluated with Kolmogorov–Smirnov or Shapiro–Wilk tests. Depending on the normality, the numerical data were expressed as mean [Standard deviation (SD)] or median [Interquartile range (IQR)]. To examine the equivalent validity of the MISA questionnaire, the relationship between the results obtained from the MISA questionnaire of the participants and other clinical measurements was examined using the Pearson correlation coefficient in those with normal distribution and the Spearman correlation coefficient (rho) in those who did not. P < 0.05 was considered statistically significant. Internal consistency of the questionnaire was evaluated with Cronbach α. The test–retest reliability of the MISA questionnaire was tested with a 2-way random effect intra-class correlation coefficient (ICC) at a 95% CI. Intolerance, according to the physician, was accepted as the gold standard. The agreement between intolerance according to the physician and intolerance according to the MISA questionnaire was made by Kappa analysis.

## Results

### Demographic and clinical features of the study population

Of the 130 patients who participated in the study, 105 (80.8%) were female, the mean age was 55.8 ± 10.50 years, and the mean disease duration was 9.9 ± 7.42 years. Some patient characteristics are presented in Table 1, and comorbidities are in Supplementary Table 2.

**Table 1:** Demographic and clinical characteristics of all patients

Variables	N:130
Age, years, (mean ± SD)	55,8 (10,50)
Gender, female, n (%)	105 (80,8)
BMI, (mean ± SD)	27,8 (5,26)
Age at diagnosis, years, (mean ± SD)	46,0 (12,11)
Disease duration, years, (mean ± SD)	9,9 (7,42)
Smoking status, ever, n(%)	31 (30)
Marital status, n (%)	
Single	7 (5,4)
Married	120 (92,3)
Divorced	3 (2,3)
Working status, n (%)	
Unemployed	70 (53,8)
Working	34 (26,2)
Retired	26 (20)
Education, n (%)	
Illiterate	17 (13,1)
Primary school	84 (64,6)
High school	23 (17,7)
University	6 (4,6)
Seropositivity	
(RF or CCP), n (%)	98 (76)
RF positivity, n (%)	82 (63,1)
CCP positivity, n (%)	85 (66,9)
Presence of EAM, n (%)	17/129 (13,2)
Prosthesis, n (%)	9/129 (67)
Rheumatoid nodule, n (%)	3/116 (2,6)
ILD, n (%)	4/116 (3,4)
Sjogren’s syndrome, n (%)	16 (12,3)
ESR, mm/h, (mean ± SD)	33,8 (20,87)
CRP, mg/L (mean ± SD)	9,9 (16,42)
TJC, (mean ± SD)	4,2 (5,10)
SJC, (mean ± SD)	1,5 (8,05)
Patient global, VAS, (mean ± SD)	36,6 (27,55)
Physician global, VAS, (mean ± SD)	21,2 (19,47)
Pain VAS, (mean ± SD)	39,6 (24,41)
DAS28-CRP, (mean ± SD)	3,1 (1,26)
DAS28-ESR, (mean ± SD)	3,8 (1,20)
CDAI, (mean ± SD)	11,5 (12,34)
HAQ, (mean ± SD)	0,7 (0,73)

*BMI* Body mass index, *CCP* Anti-cyclic citrullinated peptide, *CDAI* Clinical disease activity index, *CRP* C-reactive protein, *DAS* Disease activity score, *EAM* Extra-articular manifestation, *ESR* Erythrocyte sedimentation rate, *HAQ* Healt assesment questionnare, *ILD* Interstitial lung disease, *RF* Rheumatoid factor, *SD* Standard deviation, *SJC* Swollen joint count, *TJC* Tender joint count, *VAS* Visual analogue scale

### MTX and other treatment uses

The mean MTX intake time was 79.9 ± 68.61 months, with a mean MTX starting dose of 12.0 ± 2.64 mg/week. The current MTX usage dose averaged 14.7 ± 2.56 mg/week, and the mean number of 5 mg folic acid tablets used weekly was 2.68 ± 1.13. Of the patients, 88 (%67.7) were using oral MTX, while 42 (32.3%) used subcutaneous MTX. The number of patients receiving advanced therapy was 70 (53.8%) (Table 2).

**Table 2:** Methotrexate and other treatments of the study population

MTX and other treatments	N:130
MTX Data	
MTX intake time, month, (mean ± SD)	79,9 (68,61)
MTX starting dose mg/week, (mean ± SD)	12,0 (2,64)
MTX maximum dose, mg/week, (mean ± SD)	15,0 (2,56)
MTX current dose, mg/week, (mean ± SD)	14,7 (2,56)
Folic acid dose 5 mg tablet/week, (mean ± SD)	2,7 (1,13)
Administration	
Oral, n (%)	88 (67,7)
Subcutonouse, n (%)	42 (32,3)
csDMARDs and bDMARDs usage	
Leflunomide usage n (%)	88 (67,7)
HQ usage, n (%)	30/129 (23,3)
TNFi usage, n (%)	8/129 (6,2)
Tosilicumab usage, n (%)	3 (2,3)
Abatacept usage, n (%)	3 (2,3)
Tofacitinib usage, n (%)	3 (2,3)
Barisitinib usage, n (%)	5 (3,8)
Biological treatment usage, n (%)	33 (25,4)
MTX plus csDMARD usage, n (%)	70 (53,8)

*bDMARDs* Biologic disease-modifying antirheumatic drugs, *csDAMRDs* Conventional synthetic disease-modifying antirheumatic drugs, *HQ* Hydroxychloroquine, *MTX* Methotrexate

### MISA questionnaire results

The Cronbach’s alpha value of the MISA questionnaire was 0.831, indicating good internal consistency. The Cronbach’s alpha value when each question was removed and the mean ± SD for each question are shown in Table 3. The total score of the MISA questionnaire was 1.7 ± 3.39, and the MISA-CP score was 2.6 ± 6.10.

**Table 3:** Cronbach’s alpha values of MISA and MISA-CP scores

	MISA score	Cronbach alpha If each question is omitted	MISA-CP score
Cronbach alfa:0,831			
Q1, (mean ± SD)	0,49 (0,847)	0,788	0,528 (1,254)
Q2, (mean ± SD)	0,08 (0,414)	0,817	0,131 (0,697)
Q3, (mean ± SD)	0,22 (0,696)	0,789	0,400 (1,433)
Q4, (mean ± SD)	0,32 (0,768)	0,794	0,546 (1,575)
Q5, (mean ± SD)	0,22 (0,597)	0,797	0,308 (0,979)
Q6, (mean ± SD)	0,15 (0,563)	0,819	0,277 (1,187)
Q7, (mean ± SD)	0,08 (0,320)	0,831	0,092 (0,421)
Q8, n (%)	14 (10,8)	0,828	14 (10,8)
Q9, n (%)	8 (6,2)	0,836	8 (6,2)
Q10, n (%)	4 (3,1)	0,835	4 (3,1)
MISA total score, (mean ± SD)	1,7 (3,39)		
MISA-CP score, (mean ± SD)			2,6 (6,10)

*MISA* Methotrexate intolerance and severity assessment in adults questionnaire, *MISA-CP* MISA-Cross Product

The test–retest application was conducted with 32 patients. The ICC value for test–retest reliability was 0.894, indicating excellent reliability. Additionally, the ICC value for the MISA-CP score test–retest reliability was 0.930, reflecting excellent reliability.

According to the MISA questionnaire, 55 (42.3%) patients had a MISA score ≥ 1, and 30 (23.3%) patients were most likely to have MTX intolerance after meals. Additionally, based on the MISA questionnaire, 24 (18.5%) patients had a MISA-CP score ≥ 4. According to the physician, 38 (29.2%) patients had MTX intolerance (Table 4).

**Table 4:** Data on MISA questionnare

MISA questionnare data	N:130
MTX intolerance (MISA score ≥ 1)	55 (42,3)
1-Before meals, n (%)	3/129 (2,30)
2-After meals, n (%)	30/129 (23,3)
3-Thinking, n (%)	2/129 (1,6)
1 and 2, n (%)	15/129 (11,6)
2 and 3, n (%)	3/129 (2,3)
1, 2 and 3, n (%)	4/129 (3,1)
MISA-CP score ≥ 4, n (%)	24 (18,5)
Intolerance according to physician, n (%)	38 (29,2)

*MTX* Methotrexate, *MISA* Methotrexate intolerance and severity assessment in adults questionnaire, *MISA-CP* MISA-Cross Product

According to physician intolerance, the MISA questionnaire’s sensitivity is 100%, and the specificity is 81.5%. In the analysis comparing those who were intolerant according to the physician and those who were intolerant according to the MISA questionnaire, a good agreement was found with a kappa value of 0.721 (95% CI 0,595–0,840, p < 0,001).

MISA total score and MISA-CP score showed weak correlations with visual analog scale (VAS) physician global, VAS patient global, health assessment questionnaire (HAQ), disease activity score 28 erythrocyte sedimentation rate (DAS28-ESR), DAS28-C-reactive protein (CRP), and clinical disease activity index (CDAI) (Supplementary Table 3).

### Evaluation of intolerance according to the MISA questionnaire

When the patients were divided into two groups according to the MISA questionnaire, in terms of those with MISA total scores ≥ 1 and those with MISA total scores < 1, there were no statistically significant differences in terms of age, BMI, age at diagnosis, disease duration, marital status, occupation, smoking, education, current MTX dose, folic acid use, DAS28-ESR, and DAS28-CRP (p > 0.05). Among those with MTX intolerance, 49 (89.1%) were female, while among those without MTX intolerance, 56 (74.7%) were female, and there was a statistically significant difference in gender distribution (p = 0.039). The mean HAQ was 0.54 ± 0.69 in patients without MTX intolerance and 0.99 ± 0.70 in those with MTX intolerance, which was statistically significant (p < 0.0001). The mean CDAI values were 10.52 ± 13.49 in those without MTX intolerance and 13.03 ± 10.55 in those with MTX intolerance, which was statistically significant (p = 0.048). The highest MTX doses among the patients were 14.66 ± 2.18 in those without MTX intolerance and 16.63 ± 2.93 in those with MTX intolerance, which was statistically significant (p = 0.023). The number of patients who received advanced treatment was higher in the group with MTX intolerance, and this difference was statistically significant (p = 0.014) (Supplementary Table 4).

When patients were divided into two groups according to the MISA questionnaire based on MISA-CP total score ≥ 4 (moderate-severe MTX intolerant) and MISA-CP total score < 4, there were no statistically significant differences in terms of age, gender, BMI, marital status, occupation, smoking, education, current MTX dose and duration of use, folic acid use, DAS28-ESR, and seropositivity (p > 0.05). Disease duration and disease duration ≥ 10 years were higher in the moderate-severe MTX group. There was a statistically significant difference (p = 0.011, p = 0.026, respectively). The mean DAS28-CRP values of the patients were 3.61 ± 1.29 in those with moderate-to-severe MTX intolerance and 3.05 ± 1.24 in those without moderate-severe MTX intolerance, which was statistically significant (p = 0.048). HAQ and CDAI mean values were higher in those with MISA-CP ≥ 4 and were statistically significant (p < 0.0001, p = 0.025, respectively). Finally, those who received advanced treatment were more common in the group with MISA-CP ≥ 4, and this difference was statistically significant (p = 0.028, p = 0.0042, respectively) (Supplementary Table 5).

## Discussion

Although MTX is still considered the gold standard drug in RA, its GI side effects limit its use. Scales are needed to understand these effects better and standardize them. The first scale for GI side effects of MTX was developed by Bruner et al. in 2005 in pediatric patients with JIA (5). Later, in 2011, the MISS questionnaire was developed by Butalovic et al. in patients with pediatric JIA (10). To measure MTX intolerance and severity for the first time in adults, the MISA questionnaire was developed by Deeksha et al. in 2021 [11]. The sensitivity and specificity of the MISA questionnaire were higher than those of the MISS questionnaire developed for children. In this study, we demonstrated the validity and reliability of the Turkish version of the MISA questionnaire, which was found to be valid and reliable for adults. We developed the Turkish validation of the MISA questionnaire using methods available in the literature [13]. The questionnaire was evaluated as easy to understand, quick to administer, and applicable to the participants.

In the analysis conducted to assess the internal consistency of the questionnaire, the Cronbach's alpha value was found to be 0.831, indicating high consistency compared to the original questionnaire's Cronbach’s alpha of 0.641. In another study, during the Brazilian validation of the MISS questionnaire, Cronbach’s alpha value was found to be 0.83, similar to our study [14]. The difference in Cronbach’s alpha value was thought to be attributed to the cultural adaptation.

According to the MISA questionnaire, MTX intolerance was found in 55 patients (42.3%), similar to the literature [4, 15]. It was 38.4% in the original MISA validation study [11]. The most common symptom was nausea, consistent with other studies. Another scoring system used in the original MISA validation study was the MISA-CP scoring, which measures the severity of MTX intolerance. In our study, moderate-to-severe MTX intolerance, according to MISA-CP, was found in 18.5% of the cases, similar to the 16% reported in the original MISA validation study. While no difference was observed in terms of age between those with and without MTX intolerance in this study, in previous studies, MTX intolerance was observed in younger age groups [10, 11]. However, no difference was observed in a study by Almalag H. et al., similar to our findings [16]. This is likely due to the small sample size.

There was no statistical difference in disease duration between those with and without MTX intolerance. However, in those with moderate-to-severe MTX intolerance, the age at diagnosis was younger, and the disease duration was longer, which was statistically significant (p = 0.002, 0.011, respectively). Bulatovic et al. also found an association between MTX intolerance in patients with JIA and longer disease duration (p = 0.026) [10]. The younger age at diagnosis and longer duration of disease observed in other studies may be related to moderate-to-severe MTX intolerance. However, no scoring of MTX severity was performed in these studies, highlighting the importance of scoring the severity of MTX intolerance.

In our study, MTX intolerance was higher in female patients, similar to the study by Almalag H. et al. This effect can be attributed to the gender difference in MTX pharmacokinetics mentioned in the previous study [16].

Although the MISA scores of the patients in terms of DAS28-CRP and DAS28-ESR averages were higher than those with MTX intolerance, they did not reach statistical significance. However, according to the MISA questionnaire, there was statistical significance in CDAI and HAQ, quality of life scales, mainly clinical parameters, in those with MTX intolerance. (p = 0.025, 0.0001, respectively). At the same time, CDAI, HAQ, and DAS28-CRP levels were statistically significant in patients with a MISA score ≥ 4 in our study (p = 0.025, 0.0001, 0.048, respectively). It can be thought that as the severity of MTX intolerance increases, it also impacts disease activity and quality of life scales.

It was observed that MTX intolerance increased as the maximum dose of MTX increased (p = 0.023). This increase in intolerance with higher MTX doses has also been reported in previous studies [4, 10]. However, some studies have not shown a significant difference in intolerance with the MTX dose [11, 15, 16]. In our study, no significant difference was observed in terms of the route of administration. In Bulatovic et al.’s study in patients with JIA, intolerance was found to be significant in patients receiving parenteral MTX compared to those receiving oral MTX (67.5% vs. 44.5%, p = 0.001) [10]. However, in this study, the mean dose of patients receiving subcutaneous MTX (13.5 mg/week) was reported to be higher than those receiving oral MTX (9.5 mg/week). Similar to our study, no difference was observed in studies conducted on patients with RA regarding the route of administration [11, 14, 16].

While no difference was observed in intolerance among patients receiving conventional synthetic disease-modifying antirheumatic drugs (DMARDs), those who received advanced treatment [biologic (bDMARDs) or targeted synthetic (tsDMARDs)] were statistically more common in the MTX intolerant group (p = 0.014). Similarly, those who received advanced treatment were statistically more common in the moderate-severe MTX intolerance group (p = 0.042). This association has not been observed in previous studies [16], [17]. This effect might be due to drug interactions between MTX and b/tsDMARDs.

Since our study was a validation, we could present cross-sectional patient data. In a prospective study, the relationship between the follow-up results, the follow-up of these questionnaire scores, and drug withdrawal can be revealed more clearly.

In conclusion, this study demonstrated that the MISA questionnaire is applicable, easy to use, and reproducible in our RA patients through validity and reliability tests. To the best of our knowledge, this study represents the first instance in which the MISA questionnaire has been translated into a different language, and its validity and reliability have been demonstrated. Furthermore, we explored the factors influencing MTX intolerance. MTX intolerance was more common among females, those with higher HAQ and CDAI scores, those with higher MTX doses, and those receiving advanced therapy. Also, longer disease duration, higher HAQ and CDAI values, and advanced treatment were more prevalent in patients with moderate-to-severe MTX intolerance.

## Supplementary Information

Below is the link to the electronic supplementary material.

Supplementary file1 (DOCX 35 KB)

## Data Availability

Data are available upon reasonable request by contacting the corresponding author.
