# Supplementary material for: Validation and clinical correlates of the Turkish version of the methotrexate intolerance and severity assessment (MISA) questionnaire in rheumatoid arthritis
Source: Intern Emerg Med. 2025 Jul 18;20(5):1473–9. doi: 10.1007/s11739-025-03983-7 (PMC12331811; doi:10.1007/s11739-025-03983-7)
Supplement: Supplementary file 1 — Supplementary file1 (DOCX 35 KB) [file 11739_2025_3983_MOESM1_ESM.docx]

Supplementary Tables

Table S1. Turkish version of the MISA questionnaire

Table S2. The presence of comorbidities in the study population

Table S3. MISA total score and MISA-CP score correlation analysis

Table S4. Comparison of patients with and without intolerance according to the MISA questionnaire

Table S5. Comparison of patients with MISA-CP score<4 and MISA-CP score≥4 according to the MISA questionnaire

**Table S1. Turkish version of the MISA questionnaire**

| **Yetişkinlerde Metotreksat İntoleransı ve Şiddeti Değerlendirme Anketi (YMİŞ)** | | | | | | | |
| --- | --- | --- | --- | --- | --- | --- | --- |
| Metotreksat bazı kişilerde hoş olmayan belirtilere veya yan etkilere neden olmaktadır. Aşağıda verilen sorular bu belirtileri sorgulamaktadır.  **S1-7:**Her bir soru için, hoş olmayan belirtiniz veya hissiniz yoksa , ‘**Yok’ un** altını işaretleyin, eğer hoş olmayan belirtiniz varsa sizi ne kadar etkilediğine göre **hafif, orta, şiddetlinin** altını işaretleyiniz. Aynı zamanda, cevabınız evet ise, bu hoş olmayan belirtilerin kaç gün sürdüğünü lütfen yazınız. | | | | | | | |
| **Hoş olmayan belirtiler**  **veya yan etkiler** | Yok  0 puan | Var | | | | Belirtiler haftada kaç gün sürmekte  0,5- 7 gün arası | |
|  |  | **Hafif**  (Günlük çalışabilir)  1 puan | **Orta**  (Hafif ve şiddetli arası)  2 puan | **Şiddetli**  (Günlük çalışamaz)  3 puan | |  |  |
| 1-Metotreksat aldıktan sonra mide bulantısı hissediyor musunuz? |  |  |  |  | |  | |
| 2-Metotreksat aldıktan sonra kusuyor musunuz? |  |  |  |  | |  | |
| 3-Metotreksat aldıktan sonra midenizde rahatsızlık hissediyor musunuz? |  |  |  |  | |  | |
| 4-Metotreksat aldıktan sonra iştah kaybınız var mı veya ağzınızda kötü bir tat oluşuyor mu? |  |  |  |  | |  | |
| 5-Metotreksat aldıktan sonra uyuşuk veya vücudunuzu cansız hissediyor musunuz? |  |  |  |  | |  | |
| 6-Metotreksat aldıktan sonra sinirli veya kaygılı hissediyor musunuz? |  |  |  |  | |  | |
| 7-Metotreksat aldıktan sonra ishal veya ateşiniz oluyor mu? |  |  |  |  | |  | |
| **Skor A:** | **S1+S2+S3+S4+S5+S6+S7** | | | | | | |
| **S8-S10. Lütfen aşağıdaki soruları Evet veya Hayır olmak üzere işaretleyiniz.** | | | | | **Evet**  **1 puan** | | **Hayır**  **0 puan** |
| 8-Metotreksat almayı düşünürken bile mide bulantısı hissediyor musunuz? | | | | |  | |  |
| 9-Metotreksatın hoş olmayan belirtilerini azaltmak için kusma ilacı veya başka ilaçlar alıyor musunuz? | | | | |  | |  |
| 10-Metotreksat nedeniyle ağzınızda ağrı oluyor mu? | | | | |  | |  |
| **Skor B=S8+S9+S10** | | | | |  | |  |
| **MISA Skor= Skor A+ Skor B Semptom Süresi:SS**  **MISA CP Skoru=S1xSS+S2xSS+S3xSS+S4xSS+S5xSS+S6xSS+S7xSS +S8+S9+S10** | | | | | | | |

**Table S2. The presence of comorbidities in the study population**

| Comorbidity | N:130 |
| --- | --- |
| DM, n (%) | 22/129 (17,1) |
| HT, n (%) | 52 (40) |
| CVD, n (%) | 20 (15,4) |
| MI, n (%) | 1 (0,8) |
| CHF, n (%) | 1 (0,8) |
| SVD, n (%) | 2 (1,5) |
| HL, n (%) | 1 (0,8) |
| FMS, n (%) | 7 (5,4) |
| OP, n (%) | 19 (14,6) |
| OA, n (%) | 8 (6,3) |
| Depression, n (%) | 10 (7,7) |
| Thyroid, n (%) | 23 (17,7) |
| Malignancy, n (%) | 4 (3,1) |
| Peptic ulcus, n (%) | 2 (1,5) |
| COLD, n (%) | 2 (1,5) |
| Asthma, n (%) | 9 (6,9) |
| Lung disease, n (%) | 15/126 (11,9) |
| At least one comorbidity, n (%) | 84 (64,6) |
| At least two comorbidity, n (%) | 48 (36,9) |
| CM number, (mean±SD) | 1,28 (1,37) |

CHF: Cardiac heart failure; CM: Comobidity; COLD: Chronic obstructive lung disease; CVD: Cardiovascular diseases; DM: Diabetes mellitus; FMS: Fibromiyalgia syndrome; HL: Hyperlipidemia; HT: Hypertension; MI: Myocardial infarction; OA: Osteoarthritis; OP: Osteoporosis; SVD: Serebrovascular disease

Table S3. MISA total score and MISA-CP score correlation analysis

|  | MISA score | | MISA-CP score | |
| --- | --- | --- | --- | --- |
| Variables | **r** | **p** | **r** | **P** |
| Age | -.133 | 0.133 | -.134 | 0.134 |
| BMI | 0.098 | 0.272 | 0.99 | 0.267 |
| Disease duration | 0.140 | 0.114 | 0.145 | 0.102 |
| VAS physician global | 0.261 | **0.003** | 0.266 | **0.002** |
| VAS patient global | 0.234 | **0.008** | 0.241 | **0.006** |
| SJC | 0.162 | 0.065 | 0.162 | 0.065 |
| TJC | 0.128 | 0.146 | 0.133 | 0.130 |
| DAS28-ESR | 0.181 | **0.041** | 0.187 | **0.035** |
| DAS28 CRP | 0.196 | **0.026** | 0.201 | **0.022** |
| CDAI | 0.212 | **0.016** | 0.217 | **0.013** |
| HAQ | 0.381 | **<0.001** | 0.386 | **<0.001** |

BMI: Body mass index; CDAI: Clinical disease activity index; DAS28-CRP: Disease activity score-C-reactive protein; ESR: Erythrocyte sedimentation rate; SJC: Swollen joint count; TJC: Tender joint count; VAS: Visual analogue scale

**Table S4. Comparison of patients with and without intolerance according to the MISA questionnaire**

|  | MISA Score<1  N:75 | MISA Score≥1  N:55 | P value |
| --- | --- | --- | --- |
| Age, years, (mean±SD) | 56,71 (10,55) | 54,67 (10,412) | 0,220 |
| Age<45, n (%)  Age≥45, n (%) | 8 (%10,7)  67 (89,3) | 7 (12,7)  48 (87,3) | 0,716 |
| Gender  Female, n (%)  Male, n (%) | 56 (74,7)  19 (25,3) | 49 (89,1)  6 (10,9) | **0,039** |
| Marital status, n (%)  Single  Married  Divorced | 4 (5,3)  69 (92)  2 (2,7) | 3 (5,5)  51 (92,7)  1 (1,8) | 0,950 |
| Working status, n (%)  Unemployed  Working  Retired | 39 (52)  20 (26,7)  16 (21,3) | 31 (56,4)  14 (25,5)  10 (18,2) | 0,866 |
| Smoking  Never, n (%)  Ever, n (%) | 51 (68)  24 (32) | 40 (72,7)  15 (27,3) | 0,561 |
| Education  <8 years, n (%)  ≥8 years, n (%) | 57 (76,0)  18 (24,0) | 40 (72,7)  15 (27,3) | 0,672 |
| BMI, (mean±SD) | 27,50 (5,21) | 28,57 (5,32) | 0,272 |
| BMI<30, n (%)  BMI≥30, n (%) | 50 (67,6)  24 (32,4) | 34 (64,2)  19 (35,8) | 0,688 |
| Disease duration, years, (mean±SD) | 9,38 (7,07) | 10,71 (7,86) | 0,304 |
| Disease duration  <10 years, n(%)  ≥10 years, n(%) | 36 (48,6)  38 (51,4) | 28 (50,9)  27 (49,1) | 0,800 |
| Age of diagnosis, years, (mean±SD) | 47,1 (12,58) | 44,5 (11,39) | 0,131 |
| Seropositivity (RF or CCP), n (%)  RF positivity, n (%)  CCP positivity, n (%) | 55 (74,3)  46 (61,3)  47 (63,5) | 43 (78,2)  36 (65,5)  38 (71,7) | 0,612  0,630  0,479 |
| DAS28-ESR, (mean±SD) | 3,70 (1,18) | 4,02 (1,22) | 0,114 |
| DAS28-ESR  Moderate-high, n (%)  Low-remission, n (%) | 44 (60,3)  29 (39,7) | 39 (72,2)  15 (27,8) | 0,162 |
| DAS28-CRP, (mean±SD) | 2,99 (1,25) | 3,38 (1,25) | 0,063 |
| DAS28-CRP  Moderate-high, n (%)  Low-remission, n (%) | 30 (40,5)  44 (59,5) | 30 (54,5)  25 (45,5) | 0,115 |
| HAQ, (mean±SD) | 0,54 (0,69) | 0,99 (0,70) | **0,0001** |
| CDAI, (mean±SD) | 10,52 (13,49) | 13,03 (10,55) | **0,048** |
| Folic acid usage 5mg tablet/week, (mean±SD) | 2,61 (1,01) | 2,76 (1,29) | 0,793 |
| Current MTX dose mg/week, (mean±SD) | 14,5 (2,25) | 15,18 (2,92) | 0,105 |
| MTX starting dose, mg/week, (mean±SD) | 12,03 (2,65) | 12,11 (2,65) | 0,782 |
| MTX maximum dose, mg/week, (mean±SD) | 14,66 (2,18) | 16,63 (2,93) | **0,023** |
| MTX intake time, month, (mean±SD) | 71,63 (56,81) | 91,24 (81,21) | 0,166 |
| MTX intolerance development dose mg/week, (mean±SD) | 0 | 15,21 (2,79) |  |
| MTX administration route  Oral, n(%)  Subcutaneouse, n (%) | 53 (70,7)  22 (29,3) | 35 (63,6)  20 (36,4) | 0,397 |
| At least one CM, n (%) | 48 (64) | 36 (65,5) | 0,864 |
| Advanced therapy usage, n (%) | 13 (17,3) | 20 (36,4) | **0,014** |
| MTX plus csDMARD usage, n (%) | 25 (45,5) | 17 (56,7) | 0,323 |

BMI: Body mass index; CCP: Anti-cyclic citrullinated peptide; CDAI: Clinical disease activity index; CM Comorbidity; csDMARD: conventional synthetic disease modifying antirheumatic drugs; DAS28-CRP: Disease activity score- C-reactive protein; ESR: Erythrocyte sedimentation rate; HAQ: Health assesment questionnare; MTX: Methotrexate; RF: Rheumatoid factor; SD: Standard deviation

**Table S5. Comparison of patients with MISA-CP score<4 and MISA-CP score≥4 according to the MISA questionnaire**

|  | MISA-CP<4  N:106 | MISA-CP≥4  N:24 | P değeri |
| --- | --- | --- | --- |
| Age, years, (mean±SD) | 56,2 (10,44) | 53,2 (10,59) | 0,134 |
| Age<45, n (%)  Age≥45, n (%) | 11 (10,4)  95 (89,6) | 4 (16,7)  20 (83,3) | 0,384 |
| Gender  Female, n (%)  Male, n (%) | 84 (79,2)  22 (20,8) | 21 (87,5)  3 (12,5) | 0,354 |
| Marital status, n (%)  Single  Married  Divorced | 6 (5,7)  97 (91,5)  3 (2,8) | 1 (4,2)  23 (95,8)  0 (0) | 0,670 |
| Working status, n (%)  Unemployed  Working  Retired | 56 (52,8)  26 (24,5)  24 (22,6) | 14 (58,3)  8 (33,3)  2 (8,3) | 0,260 |
| Smoking  Never, n (%)  Ever, n (%) | 74 (69,8)  32 (30,2) | 17 (70,8)  7 (29,2) | 0,921 |
| Education  <8 years, n (%)  ≥8 years, n (%) | 79 (74,5)  27 (25,5) | 18 (75,0)  6 (25,0) | 0,962 |
| BMI, (mean±SD) | 27,79 (5,42) | 28,61 (4,59) | 0,387 |
| BMI<30, n (%)  BMI≥30, n (%) | 69 (67,00)  34 (33,00) | 15 (62,5)  9 (37,5) | 0,675 |
| Disease duration, years, (mean±SD) | 9,07 (6,70) | 13,79 (9,17) | **0,011** |
| Disease duration  <10 years, n (%)  ≥10 years, n (%) | 57 (54,3)  48 (45,7) | 7 (29,2)  17 (70,8) | **0,026** |
| Age of diagnosis, years, (mean±SD) | 47,5 (12,35) | 39,6 (8,61) | **0,002** |
| Seropositivity (RF or CCP), n(%)  RF positivity, n (%)  CCP positivity, n (%) | 80 (76,2)  68 (64,2)  70 (67,3) | 18 (75)  14 (58,3)  15 (65,2) | 0,902  0,594  0,866 |
| DAS28-ESR, (mean±SD) | 3,76 (6,70) | 4,19 (1,23) | 0,095 |
| DAS28-ESR  Moderate-high, n (%)  Low-remission, n (%) | 66 (63,5)  38 (36,5) | 17 (73,9)  6 (26,1) | 0,340 |
| DAS28-CRP, (mean±SD) | 3,05 (1,24) | 3,61 (1,29) | **0,048** |
| DAS28-CRP  Moderate-high, n (%)  Low-remission, n (%) | 45 (42,9)  60 (57,1) | 15 (62,5)  9 (37,5) | 0,082 |
| HAQ, (mean±SD) | 0,62 (0,68) | 1,23 (0,73) | **0,0001** |
| CDAI, (mean±SD) | 10,88 (12,72) | 14,68 (10,21) | **0,025** |
| Folic acid usage 5mg tablet/week, (mean±SD) | 2,63 (1,10) | 2,88 (1,26) | 0,316 |
| Current MTX dose mg/week, (mean±SD) | 14,88 (2,44) | 14,37 (3,06) | 0,509 |
| MTX starting dose, mg/week, (mean±SD) | 12,17 (2,67) | 11,66 (2,51) | 0,430 |
| MTX maximum dose, mg/week, (mean±SD) | 15,09 (2,48) | 15,00 (2,94) | 0,966 |
| MTX intake time, month, (mean±SD) | 71,98 (56,56) | 115,00 (101,09) | 0,032 |
| MTX administration route  Oral, n (%)  Subcutaneous, n (%) | 72 (67,9)  34 (32,1) | 16 (66,7)  8 (33,3) | 0,905 |
| At least one CM, n (%) | 69 (65,1) | 15 (62,5) | 0,810 |
| Advanced therapy usage, n (%) | 23 (21,7) | 10 (41,7) | **0,042** |
| MTX plus csDMARD usage, n (%) | 35 (47,3) | 7 (63,6) | 0,312 |

BMI: Body mass index; CCP: Anti-cyclic citrullinated peptide; CDAI: Clinical disease activity index; CM Comorbidity; csDMARD: conventional synthetic disease modifying antirheumatic drugs; DAS28-CRP: Disease activity score- C-reactive protein; ESR: Erythrocyte sedimentation rate; HAQ: Health assesment questionnare; MTX: Methotrexate; RF: Rheumatoid factor; SD: Standard deviation
